# Information Quality Assessment Framework for Online Dementia Care Resources

**DOI:** 10.1093/geroni/igaa057.3003

**Published:** 2020-12-16

**Authors:** Diva Smriti, Rose Ann DiMaria-Ghalili, Laura Gitlin, Aleksandra Sarcevic, Erjia Yan, Jina Huh-Yoo

**Affiliations:** 1 Drexel University, Philadelphia, Pennsylvania, United States; 2 Drexel University, Jenkintown, Pennsylvania, United States; 3 Information Science, Drexel University, Philadelphia, Pennsylvania, United States

## Abstract

Persons with dementia and caregivers can benefit from online resources. The quality and accessibility of these resources, however, can vary. We present work on the Information Quality Framework for Online Dementia Care Resources. To develop the framework, we first empirically examine resources being retrieved with query terms developed with a medical librarian. Searching one of the possible keyword combinations related to living with dementia on Google “Alzheimer AND financial planning” returned 18,900,000 results. Among the top 13 results on the first page of the search results, six were websites of government or non-profit organizations, four were for-profit companies, and three were advertisements. Out of eight unique organizations and companies, two provided support through online communities, but only one is active. The next steps include developing systematic ways to evaluate the credibility and accuracy of these resources, and search and test broader topics of dementia care resources online.

